# FRET-Based Localization of Fluorescent Protein Insertions Within the Ryanodine Receptor Type 1

**DOI:** 10.1371/journal.pone.0038594

**Published:** 2012-06-13

**Authors:** Shweta A. Raina, Jeffrey Tsai, Montserrat Samsó, James D. Fessenden

**Affiliations:** 1 Boston Biomedical Research Institute, Watertown, Massachusetts, United States of America; 2 Department of Physiology and Biophysics, Virginia Commonwealth University, Richmond, Virginia, United States of America; Baylor College of Medicine, United States of America

## Abstract

Fluorescent protein (FP) insertions have often been used to localize primary structure elements in mid-resolution 3D cryo electron microscopic (EM) maps of large protein complexes. However, little is known as to the precise spatial relationship between the location of the fused FP and its insertion site within a larger protein. To gain insights into these structural considerations, Förster resonance energy transfer (FRET) measurements were used to localize green fluorescent protein (GFP) insertions within the ryanodine receptor type 1 (RyR1), a large intracellular Ca^2+^ release channel that plays a key role in skeletal muscle excitation contraction coupling. A series of full-length His-tagged GFP-RyR1 fusion constructs were created, expressed in human embryonic kidney (HEK)-293T cells and then complexed with Cy3NTA, a His-tag specific FRET acceptor. FRET efficiency values measured from each GFP donor to Cy3NTA bound to each His tag acceptor site were converted into intermolecular distances and the positions of each inserted GFP were then triangulated relative to a previously published X-ray crystal structure of a 559 amino acid RyR1 fragment. We observed that the chromophoric centers of fluorescent proteins inserted into RyR1 can be located as far as 45 Å from their insertion sites and that the fused proteins can also be located in internal cavities within RyR1. These findings should prove useful in interpreting structural results obtained in cryo EM maps using fusions of small fluorescent proteins. More accurate point-to-point distance information may be obtained using complementary orthogonal labeling systems that rely on fluorescent probes that bind directly to amino acid side chains.

## Introduction

In structural studies of proteins using cryo electron microscopy, fusions of fluorescent proteins have been used to localize primary structure elements to cryo EM maps of large protein complexes. In these structural maps, the small fusion protein appears as a “bulge” of density within the larger protein, which is often interpreted as the location of the fusion site. This method has been used to localize specific domains in protein complexes such as viral capsids or heteromultimeric GTPases [1], [2], [3], [4]. This innovative technique has been used extensively in sequence localizations within the cardiac ryanodine receptor isoform (RyR2), a large (subunit Mr∼560 kDa) homotetrameric intracellular Ca^2+^ channel complex that plays an intrinsic role in cardiac muscle excitation contraction coupling. Many RyR2 primary sequence elements have been localized to the “clamp domains”, structures located in the corners of the RyR homotetramer. These sequence elements include positions 1366 [5] and 1874 [6], which are located in regions of high sequence divergence between the three RyR isoforms. Other positions localized to the clamp region using this technique include positions 437 [7] and 2367 [8], located within clusters of mutation sites that can lead to cardiac muscle disease. Finally, both the N-terminus of the type 3 RyR [9] and a regulatory phosphorylation site at position 2808 of RyR2 [10] have been localized to the clamp region.

While small protein fusions combined with cryo EM microscopy have yielded important structural information about RyR2, some of these findings are at variance with a recent study [11] that described the atomic structure of a 559 amino acid N-terminal fragment from RyR1, the skeletal muscle RyR isoform. The size and shape of this fragment was sufficient to enable its precise docking to a structure that surrounds a hollow vestibule within the cytoplasmic “foot” portion of RyR1 [11]. However, previous cryo EM studies using either docking of N-terminal crystal structures [12], [13] from the structurally similar inositol trisphosphate receptor [14] or localization of protein fusions at the N-terminus of RyR3 [9] or after amino acid position 437 of RyR2 [7] suggested that this RyR N-terminal domain was located in the clamp domains which are ∼100 Å from the location determined by docking the RyR1 crystal structure [10]. The reason for these divergent localizations is not known although it has been suggested [10] that the size of the inserted protein combined with the length of the glycine-rich linkers used to tether the protein to the RyR in the cryo EM studies may contribute to a significant difference in the position of inserted protein relative to its insertion point in the RyR.

**Figure 1:**
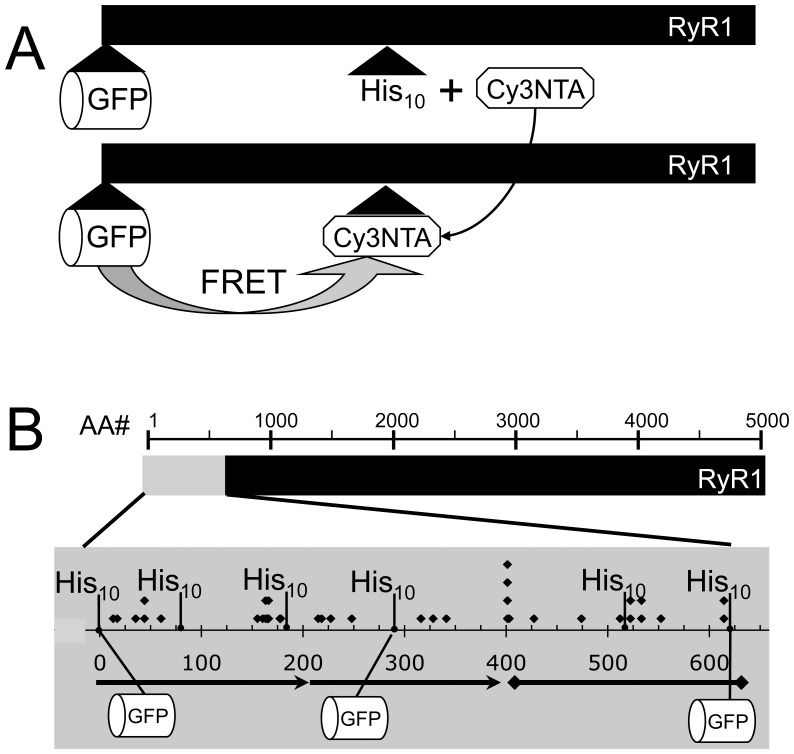
FRET-based method and GFP/His_10_ tag insertion sites used for structural analysis of RyR1. (A) Cy3NTA site-specifically binds to a His_10_ tag inserted within the primary structure of RyR1 (black bar; top) resulting in FRET from a nearby fused GFP fluorescent donor (bottom). The FRET efficiency is indicative of the proximity of the donor and acceptor fluorophores within RyR1. (B) Primary structure of RyR1 (black bar) and the N-terminal functional domain (gray bar) are indicated. Positions of GFP and His_10_ tag insertions, malignant hyperthermia mutation sites (diamonds), as well as the location of the beta sheet (arrows) and alpha helical (diamond-flanked line) subdomains [11], [21] are shown.

To understand the spatial relationship between the location of the center of mass of the fused FPs and their insertion sites within RyR1, we utilized a cell-based FRET method to probe the structure of GFP-RyR1 fusion proteins. This method relies on a Cy3/bis-nitrilotriacetic acid (NTA)/Ni^2+^ conjugate (termed Cy3NTA) that can be targeted specifically to poly-histidine “tags” engineered into RyR1. Cy3NTA can then undergo energy transfer with green fluorescent protein (GFP) fused into the primary structure of RyR1 (Fig. 1A). The FRET efficiency provides an indication of the relative proximity of these two fluorophores within RyR1.

FRET from GFP fused at each of 3 positions within the N-terminal 620 amino acids of RyR1 was measured to each of 6 different His_10_ tags placed throughout the primary sequence of this region. These FRET results were then used to triangulate each of the fused GFPs relative to the X-ray crystal structure of the N-terminal RyR1 fragment [11]. Finally, the resulting model of the N-terminal crystal structure and triangulated GFPs was docked to a cryo EM map of RyR1 [15] and the results compared with previous localizations of fused fluorescent proteins within the RyR using cryo EM techniques [9], [16].

## Results

### Experimental Approach

GFP and His_10_ tags were introduced into each of three structural sub-domains predicted by X-ray crystallography [11], [17], [18] (Fig. 1B). Thus, a set of constructs was created with GFP fused to position 1 of RyR1, a modification which affects neither orthograde nor retrograde signaling with the Ca_V_1.1 channel during EC coupling [9], [19]. A second set of constructs contained GFP fused in the middle structural subdomain at position 291. Finally, a third set of constructs was created with GFP fused at position 620, which is located beyond the crystallized area but lies at the C-terminal end of a contiguous series of alpha helices predicted by secondary structure analysis [20], [21]. FRET acceptor binding sites were engineered by inserting His_10_ tags either at positions 2, 76, 181, 290, 519 or 619. Constructs were named according to the positions of the GFP and His_10_ tag insertion sites. For example, construct GFP^291^His^519^ contained GFP and a His_10_ tag inserted after residues 291 and 519 of wtRyR1, respectively.

### Functional Testing of His-tagged GFP-RyR1 Fusion Constructs

**Figure 2:**
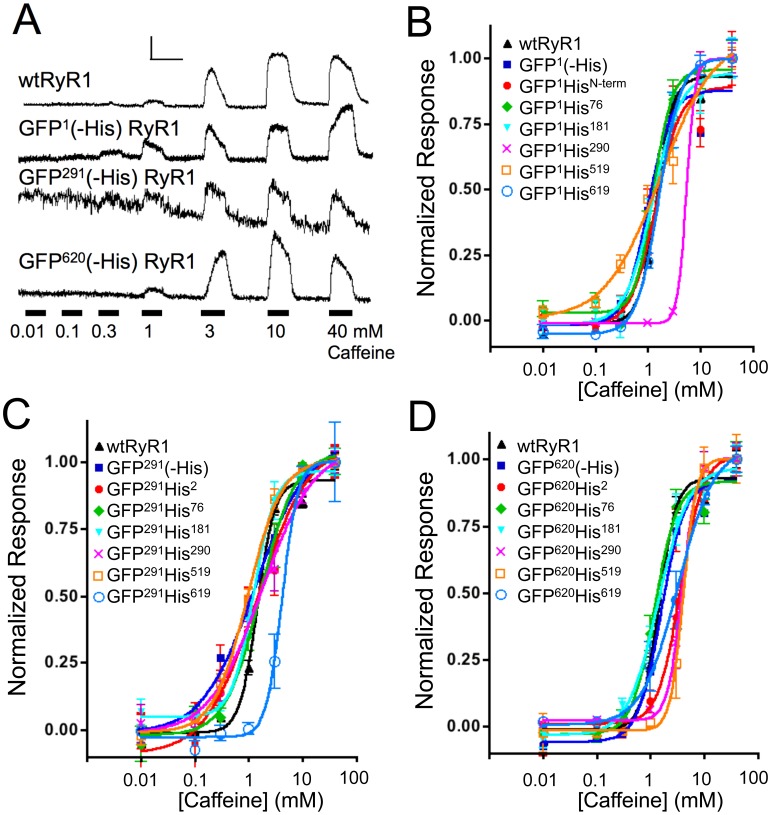
Functional analysis of His-tagged GFP-RyR1 fusion constructs. (A) Caffeine-induced Ca^2+^ transients were measured using Fluo-4-based intracellular Ca^2+^ imaging for HEK-293T cells expressing the indicated GFP-RyR1 fusion constructs. A graded series of caffeine concentrations were perfused as indicated (black bars). Individual representative traces indicate changes in Fluo-4 fluorescence normalized to resting fluorescence (F/F_0_). Calibration bar = 0.5 F/F_0_ ratio units vs. 50 sec. (B-D) Normalized caffeine dose response curves for His-tagged constructs containing GFP fused to position 1 (B), position 291 (C) or position 620 (D) of RyR1. Individual data points represent mean +/− S.E.M.

All His-tagged GFP-RyR1 fusion proteins exhibited characteristic GFP fluorescence when expressed in HEK-293T cells (data not shown) and all constructs were expressed as full-length proteins, as confirmed using Western blot analysis (Fig. S1). In addition, all constructs released Ca^2+^ in response to the RyR1 agonist, caffeine (Fig. 2). Constructs containing GFP at positions 1, 291 or 620 but lacking a His_10_ tag (Fig. 2A) had similar EC_50_ values for caffeine activation (1.03, 1.15 and 1.57 mM respectively) (Fig. 2B–D) compared to wtRyR1 expressed in HEK-293T cells (EC_50_ = 1.43 mM). All GFP-RyR1 fusion proteins containing His_10_ tags were also functional and the majority of these constructs had EC_50_ values similar to wtRyR1 (Fig. 2B–D). Only GFP^1^His^290^, GFP^291^His^619^, GFP^620^His^2^, and GFP^620^His^290^ had significantly higher EC_50_ values compared to wtRyR1 although these changes were modest (less than 4-fold) (Table 1). Untransfected HEK-293T cells did not release Ca^2+^ in response to caffeine (data not shown).

**Table 1:** Summary of EC_50_ Values for Caffeine Activation of His-tagged GFP-RyR1 Fusion Constructs.

Construct	EC_50_ (mM)^a^	95% C.I. (mM)^b^	*N* c
wtRyR1	1.43	1.26–1.62	94
GFP^1^ Series			
GFP^1^(-His)	1.03	0.55–1.91	13
GFP^1^His^N-term^	1.71	1.29–2.26	23
GFP^1^His^76^	1.37	0.93–2.02	7
GFP^1^His^181^	1.23	0.68–2.22	6
GFP^1^His^290^	5.01^d^	4.03–6.24	18
GFP^1^His^519^	1.35	0.89–2.04	21
GFP^1^His^619^	1.95	1.60–2.38	17
GFP^291^ Series			
GFP^291^(-His)	1.15	0.68–2.14	12
GFP^291^His^2^	1.10	0.59–2.05	14
GFP^291^His^76^	1.59	1.08–2.32	11
GFP^291^His^181^	1.27	0.90–1.81	13
GFP^291^His^290^	1.41	0.87–2.28	20
GFP^291^His^519^	0.93	0.71–1.22	22
GFP^291^His^619^	4.02^d^	2.86–5.67	11
GFP^620^ Series			
GFP^620^(-His)	1.57	1.05–2.36	20
GFP^620^His^2^	3.20^d^	2.38–4.21	29
GFP^620^His^76^	1.23	0.89–1.69	19
GFP^620^His^181^	1.05	0.76–1.46	16
GFP^620^His^290^	3.21^d^	2.24–4.58	12
GFP^620^His^519^	3.06	1.86–5.04	6
GFP^620^His^619^	2.07	1.10–3.87	14

a Mean EC_50_ values for caffeine activation of the indicated constructs.

b 95% confidence interval of the mean EC_50_ value.

c Number of measurements.

d EC_50_ value significantly changed relative to wtRyR1 (*p*<0.05).

A column-binding assay [22] was used to verify surface exposure of the inserted His_10_ tags (Fig. S2). Constructs with GFP at position 1 and His_10_ tags at either the N-terminus or at positions 76, 181, 290 or 519 all bound to an NTA-agarose column whereas constructs lacking a His_10_ tag did not. This finding indicates that these inserted His_10_ tags were accessible to Ni^2+^/NTA-agarose and thus should bind the FRET acceptor, Cy3NTA, which interacts with His_10_ tags via the same mechanism [23].

### FRET Measurements

**Figure 3:**
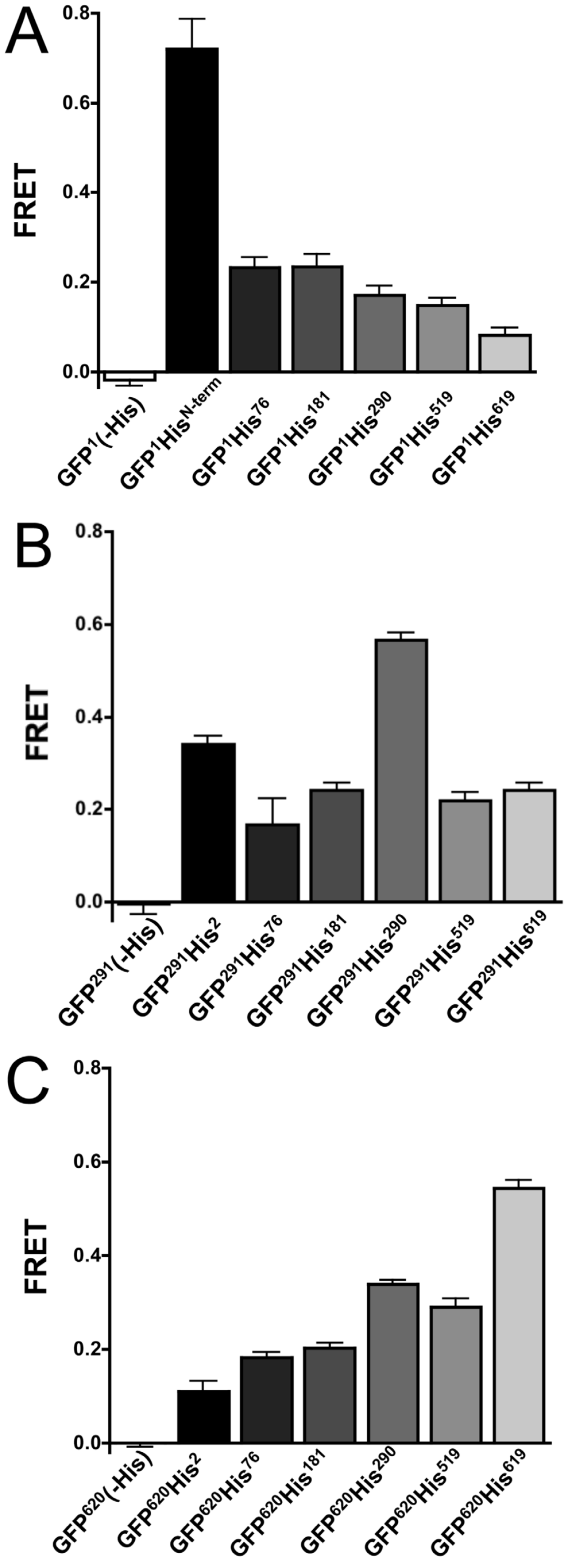
FRET analysis of His-tagged GFP-RyR1 fusion constructs. (A–C) FRET efficiencies measured from His-tagged constructs containing GFP fused to position 1 (A), position 291 (B) or position 620 (C) of RyR1 expressed in HEK-293T cells. Data points represent mean FRET efficiency +/− SEM for the indicated constructs determined from recovery of donor fluorescence after acceptor photobleaching as described in Methods.

Energy transfer measurements were performed on all GFP-RyR1 fusion proteins expressed in HEK-293T cells (Fig. 3, Table 2). No energy transfer was detected within constructs lacking a His_10_ tag (Fig. 3A–C). In contrast, the highest FRET efficiency levels observed in this study were between GFP donors and Cy3NTA acceptors targeted to His_10_ tags placed adjacent to the GFP insertion sites (*E* = 0.66, 0.57, and 0.54 for GFP^1^His^N-term^, GFP^291^His^290^ and GFP^620^His^619^, respectively, Fig. 3A–C).

**Table 2:** Summary of FRET efficiency values for His-tagged GFP-RyR1 fusion constructs.

Construct	*N* a	FRET (Observed)^b^	FRET (Predicted)^c^	
*GFP^1^ Series*				
GFP^1^(-His)	149	−0.02	*N/A*	
GFP^1^His^N-term^	93	0.66	0.59	
GFP^1^His^76^	80	0.23	0.62	
GFP^1^His^181^	52	0.23	0.25	
GFP^1^His^290^	91	0.17	0.27	
GFP^1^His^519^	106	0.14	0.16	
GFP^1^His^619^	64	0.08	N/A	
*GFP^291^ Series*				
GFP^291^(-His)	45	−0.01	*N/A*	
GFP^291^His^2^	40	0.34	0.31	
GFP^291^His^76^	20	0.16	0.11	
GFP^291^His^181^	61	0.25	0.32	
GFP^291^His^290^	42	0.57	0.62	
GFP^291^His^519^	57	0.19	0.19	
GFP^291^His^619^	82	0.24	*N/A*	
*GFP^620^ Series*				
GFP^620^(-His)	133	0.00	GFP^620^	GFP^620 alt^
GFP^620^His^2^	26	0.11	0.11	0.16
GFP^620^His^76^	75	0.18	0.28	0.20
GFP^620^His^181^	51	0.21	0.21	0.24
GFP^620^His^290^	81	0.33	0.33	0.30
GFP^620^His^519^	42	0.29	0.29	0.81
GFP^620^His^619^	53	0.54	*N/A*	

a Number of measurements.

b Mean energy transfer values observed in the FRET experiments.

c FRET efficiency values predicted after docking the model comprised of the atomic structure of the N-terminal domain and triangulated GFPs to the cytoplasmic vestibule location in the cryo EM structure of RyR1 as indicated in Figure 6. The 2 sets of values for the GFP620 series of constructs represent predicted FRET to GFP620 placed at either of the two indicated positions shown in Figure 6.

For constructs with GFP fused at position 1 (Fig. 3A), equal FRET efficiencies (*E* = 0.23) were measured when Cy3NTA was targeted to His_10_ tags at positions 76 and 181. The FRET efficiency from GFP at position 1 to Cy3NTA bound to position 290 was slightly lower (*E* = 0.17) and the FRET efficiency decreased further when Cy3NTA was targeted to positions 519 and 619 (*E* = 0.14 and 0.08, respectively).

FRET efficiency measured from GFP at position 291 was highest to Cy3NTA targeted to a His_10_ tag at position 2 (*E* = 0.34) whereas energy transfer efficiencies from GFP^291^ to Cy3NTA bound to His_10_ tags at positions 76, 181, 519 and 619 were more uniform with values of 0.16, 0.25, 0.19 and 0.24 respectively (Fig. 3B).

FRET efficiencies measured from GFP at position 620 (Fig. 3C) were highest to Cy3NTA bound to His_10_ tags at positions 291 and 519 (*E* = 0.33 and 0.29, respectively). FRET efficiencies to Cy3NTA targeted to His_10_ tags at positions 76 and 181 were roughly equivalent (*E* = 0.18 and 0.21 respectively) whereas the measured FRET efficiency was lowest to Cy3NTA bound to a His_10_ tag at position 2 (*E* = 0.11).

### Calibration of FRET Measurements

**Figure 4:**
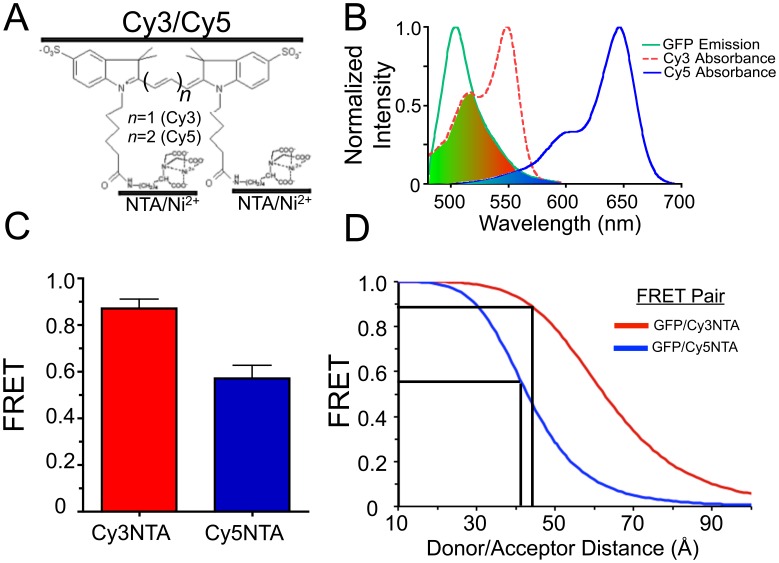
Calibration of FRET measurements using Cy5NTA. (A) Predicted structures of Cy3NTA and Cy5NTA. The number of methine groups in each compound is indicated (*n*). (B) Normalized GFP emission spectrum (green, λ_ex_ = 476 nm), as well as Cy3NTA (red) and Cy5NTA (blue) absorbance spectra. Shaded regions indicate areas of spectral overlap. (C) FRET efficiencies measured within GFPHis_10_ using either 2 µM Cy3NTA (red) or 2 µM Cy5NTA (blue) as a FRET acceptor. Values represent mean +/− S.E.M. (D) Donor-acceptor distances within GFPHis_10_ determined from theoretical FRET curves derived from the R_0_ for either GFP/Cy3NTA (R_0_ = 62.5 Å; red curve) or GFPCy5NTA (R_0_ = 42.9 Å; blue). Black lines indicate observed FRET values from panel (C) and corresponding donor/acceptor distances for each FRET pair.

To correlate these FRET measurements with donor/acceptor distances, a second FRET acceptor comprised of Cy5 coupled to 2 NTA/Ni^2+^ groups (Cy5NTA; Fig. 4A) was used. The absorbance spectrum of Cy5NTA exhibited a smaller overlap with the emission spectrum of GFP, relative to Cy3NTA (Fig. 4B) resulting in a shorter Förster distance (R_0_) of 42.9 Å compared to Cy3NTA (62.5 Å). *In vitro* FRET measurements (Fig. S3) revealed that both compounds could bind to His_10_-tagged GFP (GFPHis_10_), resulting in quenching of GFP fluorescence via FRET. However, Cy5NTA was a less efficient FRET acceptor with GFP (*E*  = 0.54) compared to Cy3NTA (*E* = 0.88) (Fig. 4C), a finding consistent with the relative R_0_ values of the two FRET pairs.

These energy transfer efficiencies corresponded to donor-acceptor distances of 44.6 Å and 41.8 Å for the GFP/Cy3NTA and GFP/Cy5NTA FRET pairs, respectively (Fig. 4D). These distances diverged when higher order binding stoichiometries were considered (Table S1) which confirms previous reports demonstrating 1∶1 binding of NTA-based fluorophores to poly-histidine tags [23], [24], [25]. The average of these distances (43.2 Å) was 18.2 Å longer than the predicted distance from the chromophoric center of GFP to its N-terminus, as derived from X-ray crystallographic data [26]. We attributed this difference to the physical dimensions of the His_10_ tag itself as well as the Cy3NTA donor, both of which most likely obscure the location of the His_10_ tag insertion point at the N-terminus of GFP. However, the use of this second FRET acceptor demonstrated that these FRET measurements reflect the distance between the donor and acceptor fluorophores while also providing an indication of the accuracy of these measurements.

### Triangulation of GFP Insertions

**Figure 5:**
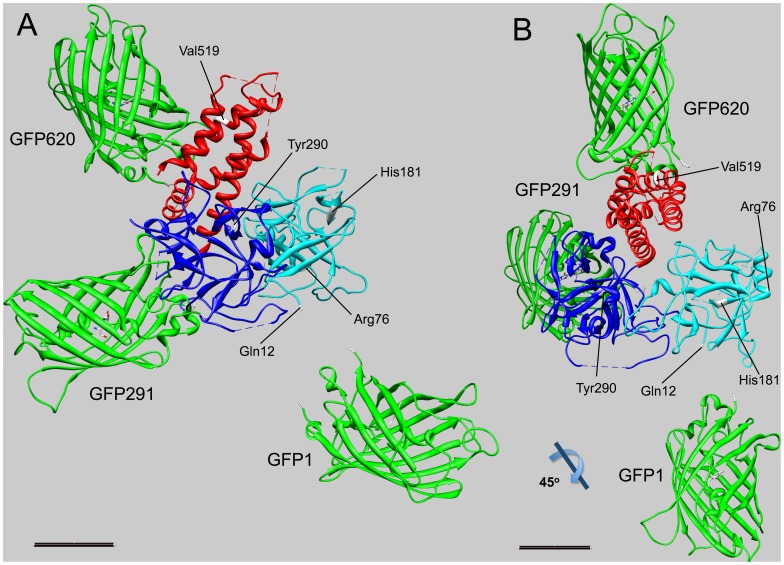
Triangulation of GFP insertions relative to the crystal structure of an N-terminal RyR1 fragment. (A) Overall view of the complex indicating the locations of the inserted GFPs and His_10_ tags. The two individual beta sheet subdomains are indicated in cyan (amino residues 12–204) and dark blue (residues 205–394), respectively. The alpha helical subdomain (residues 395–532) is indicated in red. His_10_ tag insertion sites are colored white on the ribbon depiction of the crystal structure. The X-ray crystal structure of GFP [26] inserted at each site is indicated in green. (B) The complex rotated 45° relative to the view in (A) along the indicated axis is shown. Scale bars, 20 Å.

To compare these FRET results in the context of the atomic structure of a 559 amino acid fragment from the RyR1 N-terminal domain [11], the physical locations of GFP inserted at positions 1, 291 and 620 were triangulated (see Methods) (Fig. 5). The N-terminally fused GFP was triangulated from distances measured from the His_10_ tag insertion sites at the N-terminus as well as positions 76, 181, and 290. Distances derived from FRET measurements involving position 519 did not converge with triangulations based on FRET measurements to the other positions and thus were not considered in the localization of the N-terminal GFP (nor the other GFP fusions) (see Discussion). The C-terminus of the triangulated GFP appeared to be approximately 19 Å from the alpha carbon of glutamine 12, the first residue within the crystal structure of the N-terminal RyR1 domain. The distance from the alpha carbon of glutamine 12 to the furthest end of the GFP barrel was approximately 58 Å.

GFP insertions at position 291 and 620 were also triangulated from distances to the N-terminus of RyR1 as well as positions 76, 181 and 290 (Fig. 5). The N- and C-terminal attachment points of GFP inserted at position 291 appeared to be ∼25 Å from its insertion point. GFP at position 620 could also be triangulated despite the fact that its insertion site in RyR1 lies outside the crystallized area of this domain.

### Placement of GFP Insertions within the Cryo EM Structure of RyR1

**Figure 6:**
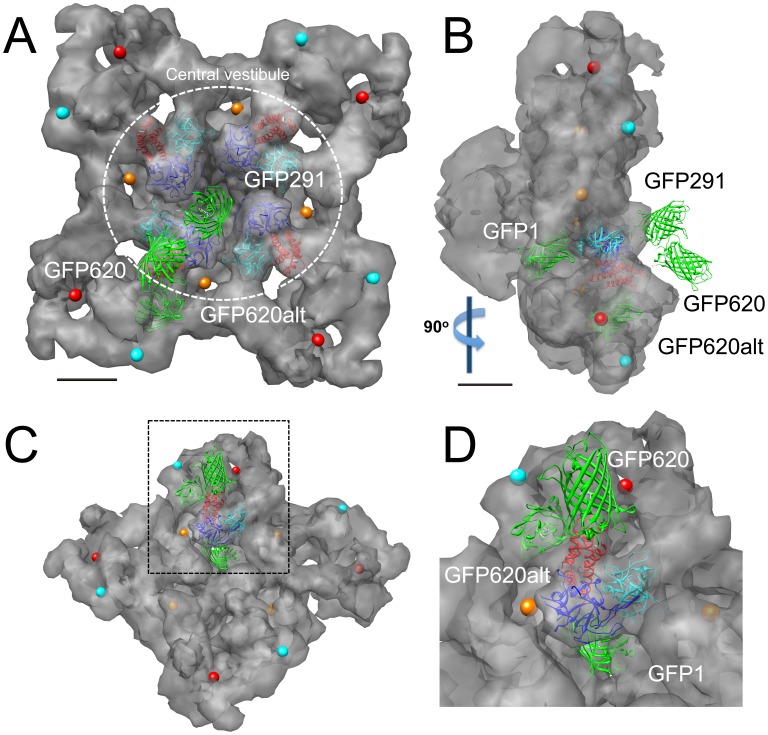
Docking of RyR1 N-terminal crystal structure and triangulated GFPs to the RyR1 cryo EM map. (A) Cryo EM structure of RyR1 (gray) viewed from the “top” (i.e. the cytoplasmic side that would face the T-tubule membrane *in situ*). Crystal structure of an N-terminal RyR1 fragment [11] is docked to a central location that forms a cytoplasmic vestibule located beneath the area indicated by the dotted circle. The positions of the GFPs (in green) inserted at the indicated positions relative to the crystal structure of the N-terminal domain docked to this position are indicated. Colored dots depict previously published localization sites of GST fused to the N-terminus of RyR3 (orange and red; [9]), as well as CFP fused to position 626 of RyR2 (cyan; [16]). (B) Side view of the cryo EM structure of RyR1 rotated 90° relative to panel A as indicated. (C) Oblique view of the cryo EM structure of RyR1 with the docking to the cytoplasmic vestibule location. (D) Magnified view of this docking from the dotted box in (C). GFP620 and GFP620alt refer to two potential localizations of GFP fused to position 620 discussed in the text. GFP at position 291 is removed to more clearly depict the locations of the X-ray crystal structure of the N-terminal RyR1 fragment as well as the other GFP fusions.

The complex consisting of the crystal structure of the N-terminal RyR1 fragment and the triangulated GFPs was docked to the cryo EM structure of RyR1 at a cytoplasmic “vestibule” proximal to the 4-fold symmetry axis of the channel (Fig. 6A,B). After docking, FRET from donor/acceptor sites between all subunits was considered and the position of the inserted GFPs adjusted as required in order to provide triangulation coordinates of these insertions that best matched our experimental FRET data (see Methods). The comparison of FRET levels determined experimentally and predicted FRET based on the location of the GFPs docked to the 3D structure of RyR1 is indicated in Table 2.

GFP fused at the N-terminus projected into a central cavity within RyR1 that is part of the “vestibule” formed by the 4 N-terminal domains (Fig. 6C,D). The chromophoric center of this GFP was 101 Å from the position of glutathione-S-transferase (GST) fused to the N-terminus of RyR3 (red dot) as determined using cryo EM microscopy [7]. However, a secondary difference density reported in that study (Fig. 6, orange dot) is as close as 25 Å to our localization of the N-terminally fused GFP. Thus, the cryo EM based localization of N-terminally fused GST to this secondary difference density is more consistent with our results and most likely represents the true location of GST fused to this position.

GFPs fused at positions 291 and 620 projected above the surface of the protein (Fig. 6B). The chromophoric center of GFP at position 620 at this location was 93 Å from a FRET-based localization of cyan fluorescent protein (CFP) fused at position 626 (Fig. 6, cyan dot) [16]. However, an alternative localization of GFP fused at position 620 (GFP620 alt, Fig. 6) could also be identified that projected into an interior location only 38 Å from the published localization of CFP fused at position 626 in RyR2 [16]. Thus, this alternative localization of GFP fused at this position is more consistent with this previous study.

## Discussion

### FRET-Based Method

The Cy3NTA labeling system employed in this study enabled the targeting of small fluorophores to specific locations within RyR1 via insertion of His_10_ tags to these locations. Non-specific binding sites for NTA-based fluorophores on RyR1 appeared to be absent since FRET was not observed for GFP-RyR1 fusion constructs lacking a His_10_ tag and also because these constructs did not bind to an NTA-agarose column. In contrast, all His_10_-tagged positions within RyR1 could bind to the NTA-agarose column, indicating that they were all surface-exposed. In addition, FRET could be measured to all His_10_-tagged sites, thus confirming the ability of Cy3NTA to bind to each of them. While differences in Cy3NTA binding affinity to the different sites may contribute to differences in FRET, this seems unlikely since the His_10_ tag binding sites are predicted to be exposed on the surface of the atomic structure of the RyR fragment and because Cy3NTA binding affinity to His_10_ tags either *in vitro* or in cells are consistent with each other (Kd∼100 nM) ([22] and Fig. S4) Finally, through the use of a second NTA-based fluorophore, Cy5NTA, we confirmed that these FRET acceptors bind to His_10_ tags with 1∶1 stoichiometry, thus indicating that differing measured FRET efficiencies do not arise from differing Cy3NTA:His_10_ tag binding stoichiometries.

The measured FRET efficiencies were indicative of molecular distances between the donor and acceptor fluorophores targeted to RyR1. The highest FRET efficiency measured in this study was for the GFP^1^His^N-term^ RyR1 construct, where a short 5 amino acid spacer element separated the His_10_ tag and GFP. Constructs with a 12 amino acid glycine rich linker separating the donor- and acceptor-binding site (GFP^291^His^290^ and GFP^620^His^619^) had slightly lower FRET efficiency levels that were consistent with the relatively longer linker between donor and acceptor fluorophores. In addition, FRET measurements of His_10_-tagged GFP using Cy3NTA or Cy5NTA yielded donor-acceptor distances that were consistent with each other as well as the molecular dimensions of GFP.

### Triangulation of GFP Insertions

GFP fused to each of the three positions in RyR1 could be localized to a unique position in space based on FRET efficiencies measured from 4 out of the 5 donor/acceptor pair combinations. The inability of all 5 donor/acceptor pairs to converge on a particular location can potentially be attributed to several factors. First, the underlying crystal structure upon which these triangulations were based may adopt a slightly different conformation or structure within the full-length protein. This could result from structural changes related either to the activation state of the channel, RyR-associated proteins or differences in the relative orientation of the three subdomains when they form native contacts with other parts of RyR1. Second, the insertions themselves may cause local structural perturbations. While all GFP-RyR1 fusion proteins in this study released Ca^2+^ in response to caffeine and fusion of small proteins at other positions do not appear to affect RyR function (for examples, see [7], [8], [16], [27]), the possibility remains that our modifications to the primary structure of the RyR disrupt its tertiary structure. Finally, the inability of GFP triangulations to converge on all 5 donor-acceptor distances could be due to intersubunit FRET, which is dependent on the location of these sites within the RyR homotetramer. This factor was taken into account for the docking experiments and is discussed below.

With these points in mind, the position of each of the inserted GFPs was triangulated relative to the crystal structure of the RyR1 domain (Fig. 5). The chromophoric centers of the triangulated GFPs were located ∼40 Å from their insertion points (when known). Given the 45-Å length of GFP, portions of the inserted protein could be as close as 19 Å and as far as 63 Å from the insertion point. These uncertainties in the position of the fusion protein inserted within the RyR may have contributed to the discrepancies in the localization of the N-terminus of the RyR to the cryo EM structure of the protein [9], [11], [14]. The results from the present study provide a more precise estimation of these uncertainties, which can be used to interpret cryo EM and FRET-based structural determinations of the RyR that rely on these types of protein insertions.

### Docking Experiments

We docked the RyR crystal structure to the central vestibule location located near the 4-fold symmetry axis of the protein [11]. After docking the complex to this location, we accounted for potential FRET between subunits by adjusting the triangulation of GFPs at each position to yield theoretical FRET values consistent with our observed FRET measurements. This adjustment was to be expected because these central cytoplasmic locations are adjacent to each other in the 3D structure of RyR1 and thus, inter-subunit energy transfer is possible. After adjustment of the position of these FPs, we observed several different types of localizations of these inserted GFPs within the 3D map of RyR1. FPs were located either within internal cavities of RyR1 (GFP at position 1), on the surface of the protein (GFP291) or in regions of high electron density (GFP620). Thus, different types of structural insertions can potentially occur when using fluorescent proteins for either cryo EM or FRET-based studies, and these factors should be kept in mind when interpreting data from these types of experiments. However, our ability to triangulate and localize GFP fusions at positions 1 and 620 to positions previously determined by other groups [9], [16] reconciles the seemingly conflicting docking of the atomic structure of the N-terminal domain and previous cryo EM-based localizations of primary sequence elements in this region. A recent cryo EM-based study also maps GFP fused at RyR2 residue 310 to this central vestibule location [28].

### Perspective

The use of fluorescent protein fusions in either FRET-based or cryo EM-based determinations of protein structure clearly has both advantages and disadvantages. The ability to genetically target a fluorescent protein to a specific location within a large protein complex with essentially 100% labeling efficiency is a clear advantage. In addition, most of the FP fusions identified in cryo EM studies appear to be localized at the surface of the protein. These insertions most likely cause less structural disturbance relative to insertions in internal portions of the channel, which could complicate analysis of structural results obtained with either fluorescent proteins or small organic dyes. However, the disadvantage of using fluorescent protein fusions is the significant distance between the fused protein and its insertion site within a larger protein complex, which adds a layer of uncertainty when interpreting either cryo EM or FRET data. Clearly, orthogonal labeling systems are required that rely on smaller protein tags that can then act as binding sites for fluorescent probes and these systems are currently being developed and implemented in our laboratory.

## Materials and Methods

### Ethics Statement

This study used the human embryonic kidney cell line (HEK-293T) obtained from the American Type Culture Collection (ATCC). The use of these cells was approved by the Partners Institutional Biosafety Committee.

### cDNA Cloning

GFP from *Aequorea coerulescens* (Takara BIO, Mountain View, CA) was inserted at either positions 1, 291 or 620 of the full length rabbit RyR1 cDNA in the pCi mammalian expression vector (Promega, Madison, WI). To promote free rotation of GFP, glycine-rich linkers similar to those used in cryo EM studies using FP insertions (for example see [5]) were added. The protein sequence of these linkers (in italics) at each insertion point within the wtRyR1 sequence (underlined) are as follows: GFP at position 1 (GFP^1^); **GFP**-*GGGGSGGGGPAGLD*IMGD-**RyR1**, GFP^291^; **RyR1-** GRYL *GGGGSGGGG*-**GFP**-*GGGGSGGGGRYL* ALTED-**RyR1**, GFP^620^; **RyR1-** NQDL *GGGGSGGGG*-**GFP**-*GGGGSGGGGDL* ITEN-**RyR1**.

DNA segments encoding His_10_ tags were inserted into the RyR1 cDNA resulting in the following protein sequences at the insertion points: His^2^; MG(H)_10_GYRDGGE-**RyR1**, His^76^; **RyR1-** SVR(H)_10_GYRALQE-**RyR1**, His^181^; **RyR1-** ERYL(H)_10_GYLSTA-**RyR1**, His^290^; **RyR1-** TGRY(H)_10_GYRYLALT-**RyR1**, His^519^; **RyR1-** KEIV(H)_10_GYLNLLY-**RyR1**, His^619^; **RyR1-** SNQD(H)_10_GYQDLITE-**RyR1**.

Constructs with His_10_ tags adjacent to the inserted GFP had the following sequences at the His tag insertion points: GFP^1^His^N-term^; MGSS(H)_10_GSQRP-**GFP**-*GGGGSGGGGPAGLDI* MGD-**RyR1**, GFP^291^His^290^; GRY(H)_10_ *GYRGGGGSGGGG*-**GFP**-*GGGGSGGGGRYL* ALTED-**RyR1**, GFP^620^His^619^; NQDL(H)_10_ *GYRGGGGSGGGG*-**GFP**-*GGGGSGGGGDL* ITEN-**RyR1**.

All insertions were performed using PCR-based primer extension followed by confirmation of correct clones using DNA sequencing and restriction digest analysis.

### Cell Culture and Ca^2+^ Imaging

HEK-293T cells were propagated and then transfected with cDNAs using polyethylenimine as described previously [22]. Three days after transfection, changes in intracellular Ca^2+^ in response to the RyR agonist caffeine were measured at 40× magnification using 2 µM Fluo-4 as a Ca^2+^ indicator as described previously [29]. Ca^2+^ transient areas calculated using Microsoft Excel were plotted as a function of caffeine concentration and then fitted to a sigmoidal dose-response function (variable slope) to determine EC_50_ values for each individual cell. These values were then compared using a 1-way analysis of variance followed by a Dunnett’s post-test with Prism 4.0 software (Graphpad Inc., San Diego, CA). A significant difference in EC_50_ values was inferred from a *p*<0.05.

### Western Blot Analysis

HEK-293T cell pellets expressing each His-tagged GFP-RyR1 fusion construct were lysed for 10 min at 37 C in 150 mM NaCl, 50 mM HEPES (pH 7.4), 0.5% sodium deoxycholate, 0.1% sodium dodecyl sulfate (SDS), 1 U/ml benzonase, and protease inhibitors (1.04 mM AEBSF, 0.8 µM aprotinin, 40 µM bestatin, 14 µM E−64, 20 µM leupeptin, 15 µM pepstatin A). Upon addition of an equal volume of 2× sample buffer consisting of 0.5 M Tris-HCl (pH 6.8), 4.4% SDS, 20% glycerol and 2% 2-mercaptoethanol, samples were incubated at 37 C for 5 min and then between 50–100 µg of total protein for each construct were loaded onto a 6% SDS-polyacrylamide gel and the samples were electrophoresed for 2 hr at 100 V. Proteins were then transferred to polyvinylidene difluoride membranes at 100 V for 1 hr at 4C [30]. Membranes were blocked in blocking buffer, consisting of 5% nonfat milk in TBS-T (50 mM Tris-HCl (pH 7.4), 150 mM NaCl and 0.05% Tween-20) for 1 hr at 4C. Membranes were then incubated in 34C anti-RyR monoclonal antibody (Developmental Studies Hybridoma Bank, Iowa City, IA) diluted 1∶200 in blocking buffer for 1 hr at 4C followed by extensive washing and then incubation in horse radish peroxidase-conjugated goat anti-mouse secondary antibody (Sigma, St. Louis, MO) diluted 1∶2000 in blocking buffer for 1 hr at 4C. After extensive washing with TBS-T, membranes were developed for 5 min in SuperSignal West Dura Extended Duration chemiluminescent substrate (Thermo, Rockford, Il) followed by a 10 min exposure on a Kodak Image Station 4000 m PRO to detect the chemiluminescent signal.

### NTA-agarose Column Chromatography

Surface exposure of His_10_ tags inserted into GFP-RyR1 fusion proteins was determined via fractionation of whole cell lysates from HEK-293T cells expressing each indicated construct on an NTA-agarose column followed by quantification of RyR content in each fraction using a RyR-specific ELISA assay as described previously [22].

### Synthesis and Purification of FRET Acceptors

Cy3NTA and Cy5NTA were synthesized and then purified via thin layer chromatography as described previously [22]. Yields quantified spectrophotometrically (Cy3 ε_550_ = 150,000 M^−1^ cm^−1^; Cy5 ε_650_ = 250,000 M^−1^ cm^−1^) were typically 40% of starting material. Before use, a dried 10 nanomole aliquot of either compound was charged with 20 nanomoles of NiCl_2_ in water.

### FRET Imaging

Three days after transfection, HEK-293T cells expressing each His-tagged GFP-RyR1 fusion protein were imaged at 40× magnification using a Leica TCS SP5 confocal microscope (Mannheim, Germany) as described previously [22]. Briefly, cells were incubated with 200 ng/ml streptolysin O (to permeabilize the cells) and 1 µM Cy3NTA for 10 min in buffer consisting of 125 mM NaCl, 5 mM KCl, 6 mM glucose, and 25 mM HEPES pH 7.6. Initial GFP and Cy3 fluorescence levels of the cells were determined from image Z-stacks recorded from the cells. Cy3NTA was then selectively bleached by illuminating the cells with 515–560 nm light from a mercury lamp attached to the confocal microscope for 5 min (Fig. S4). GFP and Cy3 fluorescence of the cells was then re-measured and the FRET efficiency (*E*) calculated from the resulting increase in GFP fluorescence after photobleaching of Cy3NTA using:
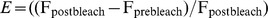
where F_prebleach_ and F_postbleach_ are GFP fluorescence intensities before and after photobleaching of Cy3NTA, respectively. F_postbleach_ values were corrected for direct photobleaching of GFP, which was determined from control experiments to be 10.2%. In some cases, FRET efficiency values were converted to intermolecular distances as described below.

In vitro *FRET measurements-* The ability of either Cy3NTA or Cy5NTA to undergo energy transfer with GFP containing an N-terminal His_10_ tag (GFPHis_10_) was determined as described previously [22]. The Förster distances (at which 50% energy transfer occurred) for either the GFP/Cy3NTA or GFP/Cy5NTA FRET pair were calculated from the GFP emission spectrum and the absorbance spectrum of Cy3/5NTA as described previously [22].

The distance from the GFP chromophore to the bound FRET acceptor was calculated using:
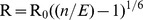
where R represents the donor/acceptor distance, R_0_ represents the Förster distance for the donor/acceptor pair, *n* represents the number of Cy3/5NTA molecules bound per His_10_ tag and *E* represents the measured energy transfer efficiency.

### Distance Measurements Relative to the RyR1 Crystal Structure

Reference distance measurements were made between the peptide bond carbonyl carbons of selected amino acid residues within the published crystal structure of an N-terminal 559 amino acid fragment of RyR1 (PDB ID 2XOA) [11] using the UCSF Chimera package from the Resource for Biocomputing, Visualization, and Informatics at the University of California, San Francisco (supported by NIH P41 RR001081) [31]. Measurements from the N-terminus of RyR1 were taken from the N-terminal nitrogen atom of residue 12, the first residue in the crystal structure.

### Triangulations of GFP Insertions

Using Chimera, spheres were centered upon the carbonyl carbon atoms of the various amino acid positions where His_10_-tags were inserted. The radii of these spheres were equivalent to the donor/acceptor distances determined from the FRET measurements. The chromophoric center of the crystal structure of GFP (PDB ID 1GFL) [26] was then placed at the intersection point of these spheres. The resulting location of all 3 GFP insertions relative to the atomic structure of the N-terminal RyR1 domain [11] was depicted using Chimera.

### Docking to the Cryo EM Structure of RyR1 and Refinement of the GFP Positions

The “Fit in Map” function in Chimera was used to dock the atomic structure of the N-terminal RyR1 domain [11] to a 10 Å resolution 3D structure of the open state of RyR1 (EMBD ID 1607) [15] at the cytoplasmic vestibule location indicated in the crystallographic report [11]. The triangulated GFPs were then initially placed in positions relative to the N-terminal domain crystal structure determined as described above. The distance from each GFP position to His_10_ positions in each of the 4 subunits was determined as described above (see *in vitro* FRET measurements) assuming 1∶1 Cy3NTA:His_10_ tag binding stoichiometry. The theoretical energy transfer rate, *k*_T_(*r*) for each of these 4 distances was determined using:
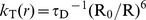
where τ_D_  =  fluorescence lifetime of the donor (GFP) in the absence of acceptor, R_0_ =  Förster distance for the GFP/Cy3NTA pair (62.5 Å) and R = distance between the given donor/acceptor pair. The individual energy transfer rates for FRET from GFP to Cy3NTA targeted to each of the 4 subunits were then summed to yield *k*_T_(*r*)_sum_ and the theoretical energy transfer efficiency for FRET between all 4 subunits was determined using:



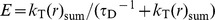



The position of the each triangulated GFP was then adjusted iteratively until this theoretical FRET value was within 20% of the measured FRET value (if possible). This process was repeated for all donor/acceptor positions and the results summarized in Table 2.

## Supporting Information

Figure S1 **Western blot analysis of His-tagged GFP-RyR1 fusion proteins.** Cell lysates expressing RyR fusion constructs with GFP at position 1 (A), 291 (B) or 620 (C) were analyzed for RyR content using Western blot analysis as described in Methods. Numbers in each panel refer to positions of molecular weight standards (in kDa). wtRyR1 and HEK-293T refers to wildtype RyR1 and untransfected cells used as positive and negative controls, respectively. Each Western blot was repeated at least 3 times with similar results.
(TIF)

Figure S2 **Determination of surface exposure of His_10_ tags inserted into GFP-RyR1 fusion proteins**. NTA-agarose fractionation of crude lysates from HEK-293T cells expressing indicated GFP-RyR1 fusion proteins. Columns were washed as indicated (dotted lines). FT = flow through. Im = imidazole. Data points indicate relative levels of RyR immunoreactivity in consecutive 120 µl fractions quantified by an RyR-specific ELISA assay (see Methods). Scale bar, 0.25 arbitrary units.
(TIF)

Figure S3 **Functional comparison of the Cy3NTA and Cy5NTA FRET acceptors**. (A) *In vitro* time-based fluorescence measurements of GFPHis_10_ incubated with indicated concentrations (in µM) of Cy3NTA (red trace) or Cy5NTA (blue). EDTA (which disrupts binding of these reagents to the His tag via chelation of the Ni^2+^ atom) was added as indicated (arrows). (B) Concentration dependence of FRET from GFPHis_10_ to either Cy3NTA (red curve) or Cy5NTA (blue) determined using *in vitro* measurements.
(TIF)

Figure S4 **Optimization of experimental conditions for cell-based FRET measurements of His-tagged GFP RyR1 fusion constructs**. (A) Timecourse of recovery of donor fluorescence from GFP^1^His^N-term^ construct expressed in HEK-293T cells after photobleaching Cy3NTA for the times indicated. FRET efficiency was quantified as described in Methods. (B) Cy3NTA concentration dependence for determining FRET efficiency via acceptor photobleaching. Data points each represent mean +/− SEM for 14 cells (A) and 8–21 cells (B).
(TIF)

Table S1 **Effect of Different Cy3/5NTA Binding Stoichiometries on Calculated Donor/Acceptor Distances.** ^a^Calculated donor/acceptor distance for GFPHis_10_ construct using either Cy3NTA or Cy5NTA as FRET acceptor. ^b^Difference in calculated donor/acceptor distances using the two FRET acceptors.
(DOCX)
