# Supplementary figures and images for: FRET-Based Localization of Fluorescent Protein Insertions Within the Ryanodine Receptor Type 1
Source: PLoS One. 2012 Jun 13;7(6):e38594. doi: 10.1371/journal.pone.0038594 (PMC3374828; doi:10.1371/journal.pone.0038594)

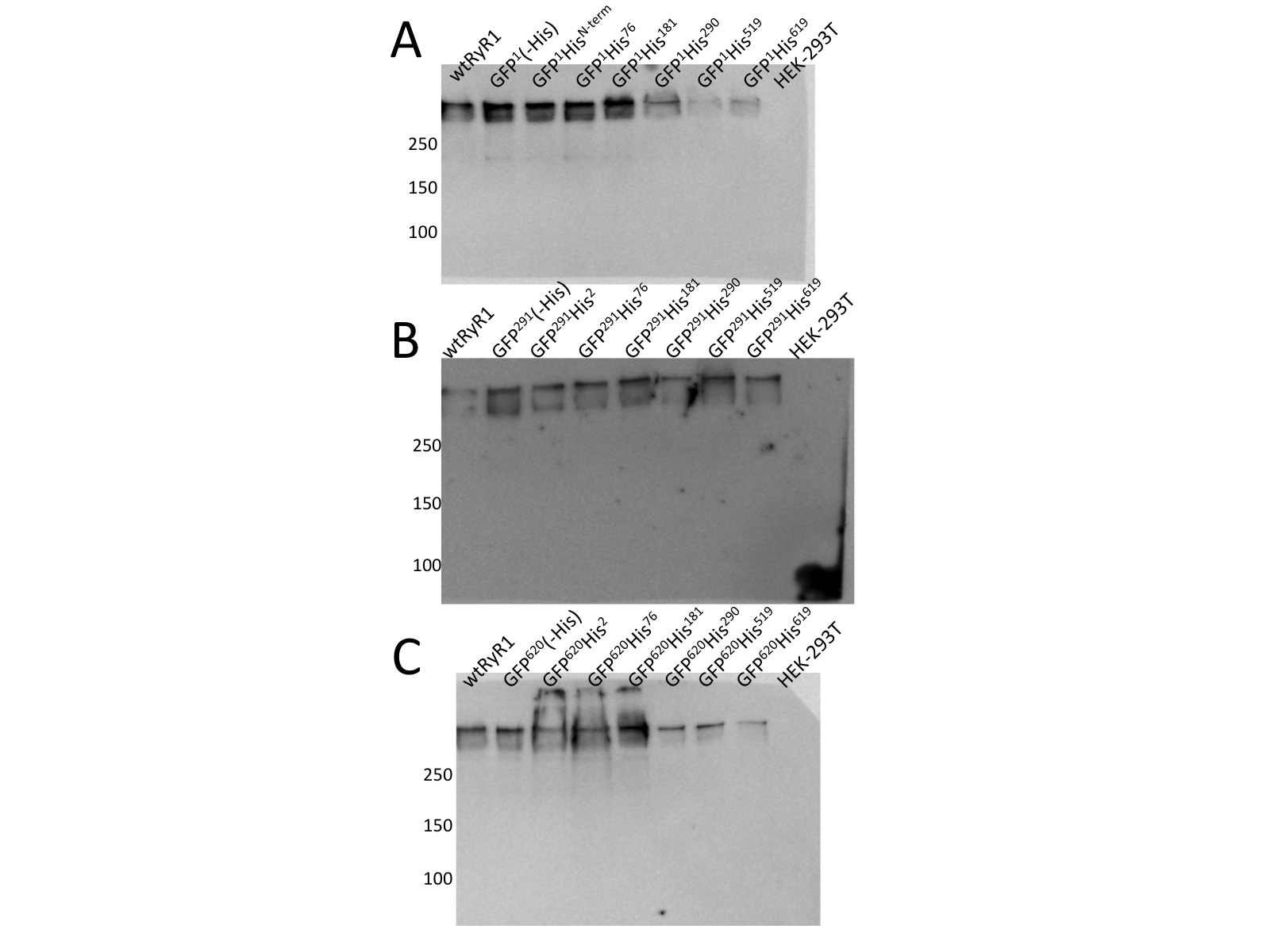

Supplement: Figure S1 — Western blot analysis of His-tagged GFP-RyR1 fusion proteins. Cell lysates expressing RyR fusion constructs with GFP at position 1 (A), 291 (B) or 620 (C) were analyzed for RyR content using Western blot analysis as described in Methods. Numbers in each panel refer to positions of molecular weight standards (in kDa). wtRyR1 and HEK-293T refers to wildtype RyR1 and untransfected cells used as positive and negative controls, respectively. Each Western blot was repeated at least 3 times with similar results. (TIF) [file pone.0038594.s001.tif]

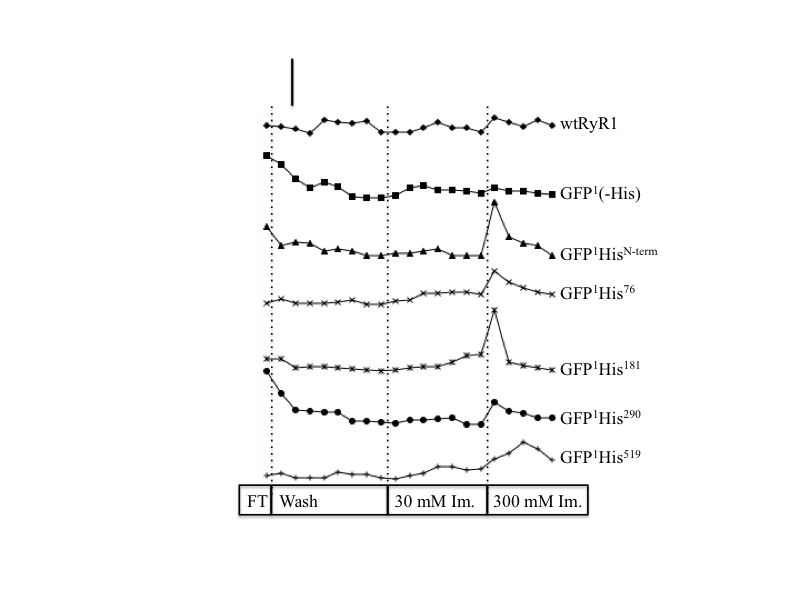

Supplement: Figure S2 — Determination of surface exposure of His10 tags inserted into GFP-RyR1 fusion proteins. NTA-agarose fractionation of crude lysates from HEK-293T cells expressing indicated GFP-RyR1 fusion proteins. Columns were washed as indicated (dotted lines). FT = flow through. Im = imidazole. Data points indicate relative levels of RyR immunoreactivity in consecutive 120 µl fractions quantified by an RyR-specific ELISA assay (see Methods). Scale bar, 0.25 arbitrary units. (TIF) [file pone.0038594.s002.tif]

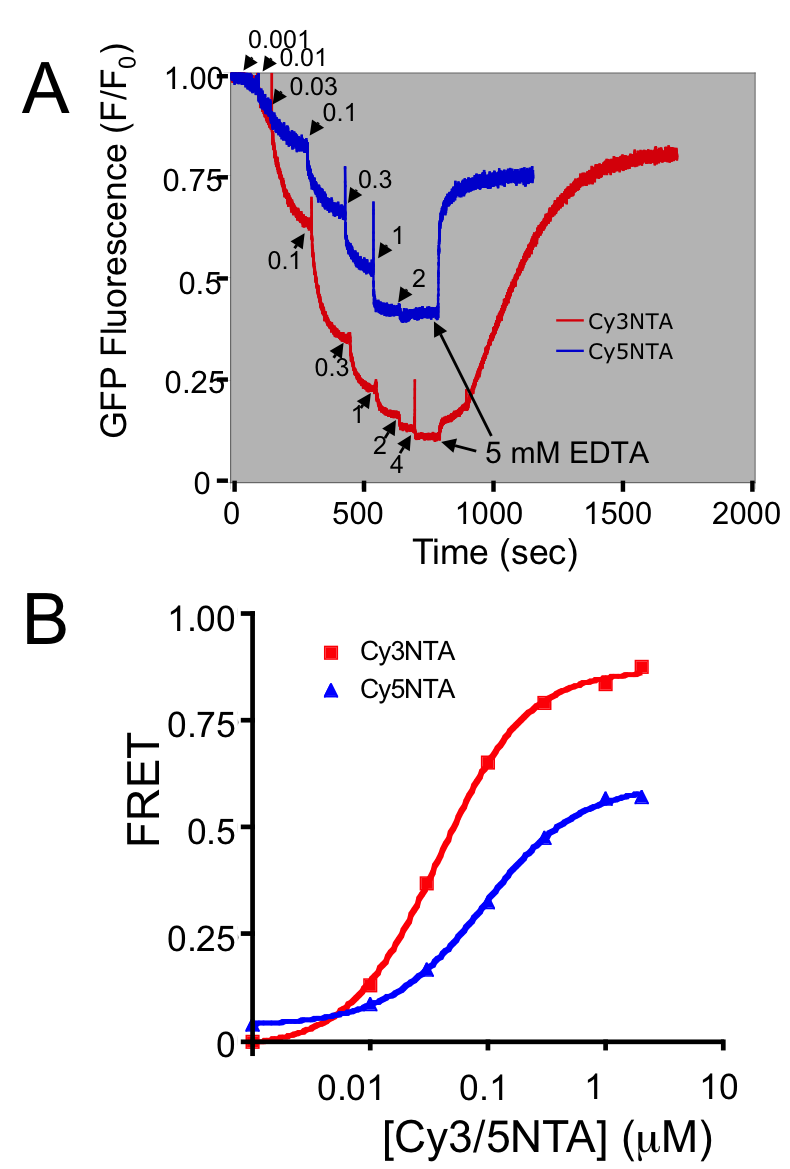

Supplement: Figure S3 — Functional comparison of the Cy3NTA and Cy5NTA FRET acceptors. (A) In vitro time-based fluorescence measurements of GFPHis10 incubated with indicated concentrations (in µM) of Cy3NTA (red trace) or Cy5NTA (blue). EDTA (which disrupts binding of these reagents to the His tag via chelation of the Ni2+ atom) was added as indicated (arrows). (B) Concentration dependence of FRET from GFPHis10 to either Cy3NTA (red curve) or Cy5NTA (blue) determined using in vitro measurements. (TIF) [file pone.0038594.s003.tif]

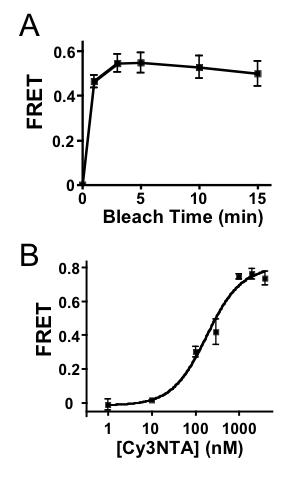

Supplement: Figure S4 — Optimization of experimental conditions for cell-based FRET measurements of His-tagged GFP RyR1 fusion constructs. (A) Timecourse of recovery of donor fluorescence from GFP1HisN-term construct expressed in HEK-293T cells after photobleaching Cy3NTA for the times indicated. FRET efficiency was quantified as described in Methods. (B) Cy3NTA concentration dependence for determining FRET efficiency via acceptor photobleaching. Data points each represent mean +/− SEM for 14 cells (A) and 8–21 cells (B). (TIF) [file pone.0038594.s004.tif]
