# Supplementary material for: FRET-Based Localization of Fluorescent Protein Insertions Within the Ryanodine Receptor Type 1
Source: PLoS One. 2012 Jun 13;7(6):e38594. doi: 10.1371/journal.pone.0038594 (PMC3374828; doi:10.1371/journal.pone.0038594)
Supplement: Table S1 — Effect of Different Cy3/5NTA Binding Stoichiometries on Calculated Donor/Acceptor Distances. aCalculated donor/acceptor distance for GFPHis10 construct using either Cy3NTA or Cy5NTA as FRET acceptor. bDifference in calculated donor/acceptor distances using the two FRET acceptors. (DOCX) [file pone.0038594.s005.docx]

| Cy3/5NTA:His_10_ tag stoichiometry | Cy3NTA  Distance^a^ | Cy5NTA  Distance^a^ | Difference^b^ |
| --- | --- | --- | --- |
| 1:1 | 44.6 Å | 41.8 Å | 2.8 Å |
| 2:1 | 64.9 Å | 50.7 Å | 14.2Å |
| 3:1 | 72.3 Å | 55.3 Å | 17.0 Å |

Table S1: Effect of Different Cy3/5NTA Binding Stoichiometries on Calculated Donor/Acceptor Distances

^a^ Calculated donor/acceptor distance for GFPHis_10_ construct using either Cy3NTA or Cy5NTA as FRET acceptor.

^b^ Difference in calculated donor/acceptor distances using the two FRET acceptors.
